# Subcellular distribution of non-muscle myosin IIb is controlled by FILIP through Hsc70

**DOI:** 10.1371/journal.pone.0172257

**Published:** 2017-02-24

**Authors:** Hideshi Yagi, Tetsuji Takabayashi, Min-Jue Xie, Kazuki Kuroda, Makoto Sato

**Affiliations:** 1 Department of Anatomy and Cell Biology, Hyogo College of Medicine, Hyogo, Japan; 2 Division of Cell Biology and Neuroscience, Department of Morphological and Physiological Sciences, Faculty of Medical Sciences, University of Fukui, Fukui, Japan; 3 Research Center for Child Mental Development, University of Fukui, Fukui, Japan; 4 United Graduate School of Child Development, Osaka University, Kanazawa University, Hamamatsu University School of Medicine, Chiba University and University of Fukui, Osaka, Japan; 5 Department of Anatomy and Neuroscience, Graduate School of Medicine, Osaka University, Osaka, Japan; Semmelweis Egyetem, HUNGARY

## Abstract

The neuronal spine is a small, actin-rich dendritic or somatic protrusion that serves as the postsynaptic compartment of the excitatory synapse. The morphology of the spine reflects the activity of the synapse and is regulated by the dynamics of the actin cytoskeleton inside, which is controlled by actin binding proteins such as non-muscle myosin. Previously, we demonstrated that the subcellular localization and function of myosin IIb are regulated by its binding partner, filamin-A interacting protein (FILIP). However, how the subcellular distribution of myosin IIb is controlled by FILIP is not yet known. The objective of this study was to identify potential binding partners of FILIP that contribute to its regulation of non-muscle myosin IIb. Pull-down assays detected a 70-kDa protein that was identified by mass spectrometry to be the chaperone protein Hsc70. The binding of Hsc70 to FILIP was controlled by the adenosine triphosphatase (ATPase) activity of Hsc70. Further, FILIP bound to Hsc70 via a domain that was not required for binding non-muscle myosin IIb. Inhibition of ATPase activity of Hsc70 impaired the effect of FILIP on the subcellular distribution of non-muscle myosin IIb. Further, in primary cultured neurons, an inhibitor of Hsc70 impeded the morphological change in spines induced by FILIP. Collectively, these results demonstrate that Hsc70 interacts with FILIP to mediate its effects on non-muscle myosin IIb and to regulate spine morphology.

## Introduction

Many actin-binding proteins, such as myosins, control actin cytoskeleton dynamics. Myosins are divided into at least twenty-four classes and have roles in numerous cellular functions, including cytokinesis, organelle transport, cell polarization, and signal transduction [1,2]. Among them, class II myosins form thick filaments and have a role in cellular contraction [2]. In the skeletal muscle, muscle type myosin II assembles and forms thick filaments, which are components of myofibrils. Contraction of muscle cells is made possible by the intercalation of actin fibers and myosin fibers. The proper assembly and quality control of myosin II is essential for the function of muscles [3–6]. Non-muscle cells contain stress fibers that resemble the myofibrils of the muscle cell, composed of bundles of actin and thick filaments of non-muscle myosin II [7–9]. This organized structure has an important role in cytokinesis and cell motility in non-muscle cells [8,10]. As these cell reactions involve dynamic actions, rapid dissociation and re-association of the stress fibers are needed. Thus, elucidating the mechanism of assembly and disassembly of the components of stress fibers is important for understanding cellular response to alterations in the circumstances of the cell. Whereas the mechanism of myosin II assembly is well investigated and the importance of chaperone proteins for the assembly of the myofibrils in the muscle has been established [11], the mechanisms underlying the disassembly of myosin II from the actomyosin complex have not been fully revealed yet.

In neurons, non-muscle myosin IIb controls the morphology of neuronal spines where excitatory synapses are made, such that inhibition of non-muscle myosin IIb leads to the elongation of spines [12–14]. We recently reported that the expression of filamin-A interacting protein (FILIP, FILIP-1) results in the alteration of the subcellular distribution of non-muscle myosin IIb in cultured cells. Specifically, FILIP leads to a shift in expression of non-muscle myosin IIb from the stress fiber-like cytoskeleton fraction to the cytosolic fraction [15]. FILIP expression also results in the elongation of spines in neurons [15]. This indicates that FILIP is involved in the dissociation of non-muscle myosin IIb from stress fiber-like structures. Furthermore, the dissociation of non-muscle myosin IIb is implicated in alterations of the spines and synaptic structure of neurons. These data establish that the binding of FILIP to non-muscle myosin IIb influences the subcellular distribution of non-muscle myosin IIb. However, the precise mechanism underlying the FILIP-mediated dissociation of non-muscle myosin IIb from stress fibers has not been fully established.

The objective of this study was to identify potential binding partners of FILIP contributing to its regulation of non-muscle myosin IIb. Here, we identify a chaperone protein, Hsc70, as a new binding partner of FILIP and present data indicating that Hsc70 has important roles in the effects of FILIP on non-muscle myosin IIb.

## Materials and methods

### Animals

All experiments were conducted in accordance with the Regulations for Animal Research at University of Fukui and the Regulations for Animal Experimentation at Hyogo College of Medicine. The Animal Research Committee at University of Fukui and the President of Hyogo College of Medicine, under the review of the Hyogo College of Medicine Animal Experiment Committee, approved these experiments. *FILIP*-knockout mice and C57BL6/J mice were maintained on a 12-hour light/dark cycle in a temperature- and humidity-controlled environment with free access to food and tap water. The morning that the presence of a vaginal plug was confirmed was defined as embryonic day 0.5 (E 0.5). Pregnant females were deeply anesthetized using sodium pentobarbital (40 mg/kg). The embryos were deeply anesthetized using hypothermia. All efforts were made to minimize suffering.

### Cell culture

COS-7 cells [16] and NIH3T3 cells [17] were cultured in Dulbecco’s Modified Eagle’s Medium (DMEM) containing 10% fetal bovine serum at 37°C in a humidified air atmosphere supplemented with 5% CO_2_.

### Construction of vectors used in this study

Full-length rat L-FILIP cDNA was amplified using polymerase chain reaction (PCR) and was inserted into the pCAGGS vector [18], which contains 3 x FLAG and IRES-GFP sequences. The PCR primers for the construction of full-length FILIP were as follows (listed 5’ to 3’): gcagatctggtgggaatgagatcacgaaatcaagg and cggatatcccttcccccctccaagaga. The control vector was the empty pCAGGS vector expressing IRES-GFP (pCAGGS IRES-GFP). The vectors expressing the truncated forms of FILIP were constructed using the KOD-plus-mutagenesis kit (Toyobo, Tokyo, Japan) based on the 3 x FLAG tagged full-length FILIP expression vector (pCAGGS FILIP IRES-GFP). The primers for the truncated forms of FILIP were as follows (listed 5’ to 3’): FILIP d248-442, gagggcgaaggacttgagcttcac and cggagtaagtcggaatgcacccag; FILIP d248-685, gagggcgaaggacttgagcttcac and caaatggccaagcacaaagccata; FILIP d934-1111, ctcttcagatgtggggcttgtgat and aggaaccacctctcttcaagaccc; FILIP d872-1111, ccaaggaatccaagacttcctca and aggaaccacctctcttcaagaccc; and FILIP d687-960, tcgcaaaagcccaaaagtgca and ttggtgtttgatttcctcgag. A schematic depicting each mutant is shown in S1 Fig. For the enhanced yellow fluorescent protein (EYFP)-tagged FILIP expression vector, the full-length rat L-FILIP cDNA was inserted into the pEYFP N1 vector (Takara Bio Inc., Kusatsu, Japan).

### Pull-down assay using glutathione S-transferase fusion proteins

The FILIP 935–1111 fragment was subcloned into the pGEX 6p-1 vector (GE Healthcare UK, Amersham Place, England). BL21 (DE3) *Escherichia coli* cells containing the vectors were incubated in LB broth, and isopropyl β-D-1-thiogalactopyranoside was used to induce protein production. The glutathione S-transferase (GST) fusion proteins were coupled to Glutathione Sepharose 4B (GE Healthcare UK). NIH3T3 cells in 10-cm dishes were lysed in 1.0 ml of lysis buffer (150 mM NaCl, 20 mM Tris-HCl (pH 7.4), 1% Triton X-100, 5 mM sodium pyrophosphate, 50 mM NaF, 10 mM β-glycerophosphate, and a proteinase inhibitor cocktail (Nacalai Tesque Inc., Kyoto, Japan)) at 4°C for 30 min. The sample was centrifuged at 15000 rpm at 4°C for 30 min to collect the supernatant. The extract was then incubated with 20 μl of glutathione-Sepharose beads containing the GST fusion FILIP protein at 4°C overnight. After the beads were washed with phosphate-buffered saline (PBS), the bound protein was eluted by boiling the beads in the sodium dodecyl sulfate (SDS) sample buffer for 5 minutes. The proteins were separated by SDS-polyacrylamide gel electrophoresis (SDS-PAGE) and were identified by the matrix-assisted laser desorption/ionization time of flight mass spectrometry (MALDI-TOF MS, Bruker Daltonics Autoflex; Bruker Daltonics K.K., Yokohama, Japan).

### DNA transfection and immunocytochemistry

COS-7 cells and NIH3T3 cells were cultured on fibronectin-coated cover glasses and then transfected with the pCAGGS FILIP IRES-GFP vector or the pCAGGS IRES-GFP control vector using FuGENE 6 transfection reagent (Roche Diagnostics, Tokyo, Japan) according to the manufacturer’s instructions. For the jasplakinolide treatment, the medium was changed to jasplakinolide-containing medium 30 minutes before fixation of the cells. After fixing the cells using 4% paraformaldehyde (PFA) in 0.1 M phosphate buffer (pH 7.2) 24 hours after transfection, we permeabilized the cells in antibody dilution buffer (PBS containing 0.25% Triton X-100 and 1% normal goat serum). To analyze the distribution of non-muscle myosin IIb, we incubated the cells in antibody dilution buffer containing a polyclonal anti-NMHCIIb antibody (1:1,000, Covance, Emeryville, CA). After washing with PBS, the signals were visualized using Alexa 568-conjugated anti-rabbit antibody (1:2,000, Invitrogen, Grand Island, NY). To analyze actin fibers, we permeabilized the cells in antibody dilution buffer and then stained them using Alexa 568-conjugated phalloidin (1:40, Invitrogen). To treat the cells with clofibric acid, we added 1 M clofibric acid in ethanol to the culture medium to a final concentration of 2 mM 4 hours after the transfection. The cells were cultured for 24 hours, fixed, and stained, as described above. The cells were analyzed using confocal microscopy (LSM 510, Carl Zeiss, Oberkochen, Germany). Non-muscle myosin IIb distribution in the cells was categorized as either having a stress fiber-like distribution or a granular distribution. “Stress fiber-like distribution” indicates that the cells had thick fibers that are longer than the diameter of the nucleus. “Granular distribution” indicates that the cells lacked thick, long fibers but had intracellular particle-like spots.

### Immunoprecipitation

COS-7 cells cultured in 6-cm dishes were lysed in 400 μl of ice-cold lysis buffer 24 hours after transfection with the plasmid vectors. After centrifugation at 15,000 rpm for 30 minutes at 4°C, 350 μl of the lysates were incubated with antibodies conjugated to 15 μl of Protein G Dynabeads (Invitrogen) at 4°C for 3 hours. The antibodies used for immunoprecipitation were as follows: 0.5 μl of polyclonal anti-NMHCIIb antibody against endogenous non-muscle myosin IIb and 0.2 μl of monoclonal anti-FLAG M2 antibody (Sigma-Aldrich, St. Louis, MO) to detect the FLAG-tagged FILIP. After the Dynabeads were rinsed three times in lysis buffer, the immunoprecipitated proteins were eluted in 1 x SDS buffer and boiled for 3 minutes. Immunoprecipitation products and cell lysates (half of the immunoprecipitation product and 7 μL of the cell lysates) were separated by SDS-PAGE and transferred onto polyvinylidene difluoride membranes (EMD Millipore Corporation). After the membranes were blocked with 5% fat-free milk in PBS containing 0.1% Tween 20, they were incubated with the following primary antibodies: rat monoclonal anti-Hsc70 antibody (1:2000; GeneTex, Hsinchu City, Taiwan), polyclonal anti-NMHCIIb antibody (1:2000), or monoclonal anti-FLAG M2 antibody (1:2000; Sigma-Aldrich Co. LLC). Membranes were then incubated with appropriate secondary antibodies coupled to horseradish peroxidase (1:2000; BD Biosciences, Franklin Lakes, NJ). The peroxidase activity was detected using enhanced chemiluminescence.

### Primary culture of piriform cortex and hippocampal neurons

The piriform cortices and the hippocampal formation were dissected from mouse brains at E17.5. The tissues were digested using 90 U of papain (Worthington Biochemical Corporation, Lakewood, NJ) in 1 ml of PBS(-) for 20 minutes at 37°C. The tissues were triturated and the dissociated cells were plated on polyethyleneimine-coated dishes in DMEM/10% fetal calf serum. After the cells attached to the dishes, the medium was changed to MACS Neuro Medium containing MACS NeuroBrew-21 (Miltenyl Biotec K. K., Tokyo, Japan), l-glutamine (2 mM), penicillin (100 U), and streptomycin (0.1 mg/ml). Hippocampal neurons were transfected with the pCAGGS FILIP IRES-GFP vector, the pCAGGS FILIP d872-1111 IRES-GFP vector, or the pCAGGS IRES-GFP vector and the pCAGGS tdTomato vector using Lipofectamine 2000 (Invitrogen) at day in vitro (DIV) 17. Piriform neurons were transfected with a pCAGGS tdTomato vector using Lipofectamine 2000 (Invitrogen) at DIV17. The cells were treated with an Hsc70/Hsp70 inhibitor (2 μM clofibric acid for 12 hours or 4 μM VER-155008 [Sigma-Aldrich] for 6 hours) and fixed using 4% PFA in 0.1 M phosphate buffer at DIV20. To calculate spine lengths, images were composed from Z-stacked images captured using a confocal microscope in ImageJ (National Institutes of Health, Bethesda, MD).

### Statistical analyses

We used Welch’s *t*-tests for statistical analyses of spine length. We used Fisher’s exact tests for statistical analyses of summarized categorized data. P < 0.05 was considered significant.

## Results

### FILIP binds to Hsc70

**Fig 1:**
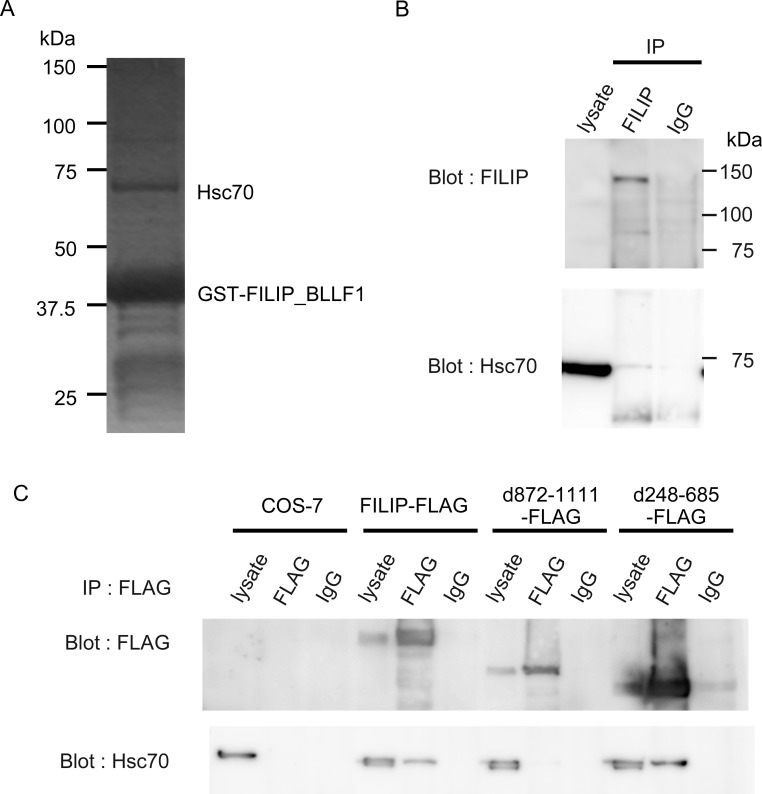
Hsc70 is a binding partner of FILIP. (A) An SDS-PAGE gel image of the co-purified proteins with a partial fragment of FILIP including the Herpes_BLLF1 domain fused to glutathione S-transferase. (B) FILIP binds to Hsc70 *in vivo*. Protein lysates were obtained from the adult mouse heart. Immunoprecipitation was performed using an anti-FILIP antibody, and blots were probed with an anti-Hsc70 antibody. (C) The Herpes_BLLF1 domain of FILIP is responsible for binding to Hsc70. The indicated vectors were transfected into COS-7 cells. Immunoprecipitation was performed using an anti-FLAG antibody, and blots were probed using an anti-Hsc70 antibody. IgG, normal mouse IgG as a negative control.

FILIP has two domains, the SMC_prok_B domain (TIGR02168) and the Herpes_BLLF1 domain (pfam05109) (http://www.ncbi.nlm.nih.gov/Structure/cdd/wrpsb.cgi) [19]. In order to investigate the mechanism by which FILIP functions, we searched for its potential binding partners. We focused on the Herpes_BLLF1 domain, whose function remains obscure. We generated a GST-Herpes_BLLF1 domain fusion protein. One candidate protein with a molecular weight of approximately 70 kDa was detected using a pull-down assay and was subsequently identified as Hsc70 using MALDI-TOF MS (Fig 1A). The binding of FILIP to Hsc70 was confirmed using immunoprecipitation (Fig 1B and S2 Fig). We investigated the importance of the Herpes_BLLF1 domain for the binding of Hsc70 to FILIP using several deletion mutants of FILIP. Deletion of amino acids 872–1111 of the FILIP Herpes_BLLF1 domain resulted in the loss of Hsc70 binding (Fig 1C).

### Deletion of the Hsc70 binding site does not influence the binding of non-muscle myosin IIb and FILIP

**Fig 2:**
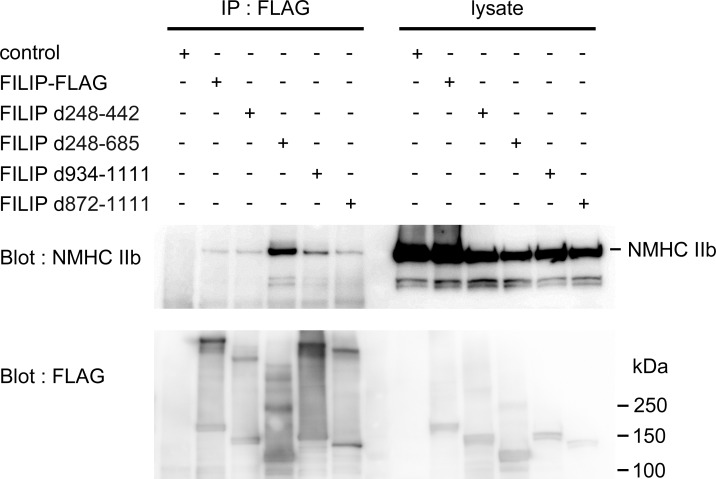
Deletion of the SMC_prok_B domain or the Herpes_BLLF1 domain of FILIP do not interfere with its binding to non-muscle myosin IIb. The indicated vectors were transfected into COS-7 cells. Immunoprecipitation was performed using an anti-FLAG antibody, and blots were probed with an anti-NMHCIIb antibody. Non-muscle myosin IIb was co-immunoprecipitated with FLAG-tagged full-length FILIP, FILIP d248-442, FILIP d248-685, FILIP d934-1111, and FILIP d872-1111. Lysates, corresponding total cell lysate fractions. Control means immunoprecipitation product or cell lysate derived from pCAGGS IRES-GFP-transfected COS-7 cells.

We investigated whether Hsc70 influences the binding of FILIP to non-muscle myosin IIb. FILIP d872-1111 co-immunoprecipitated with non-muscle myosin IIb (Fig 2). The other deletion mutants, FILIP d248-442 and FILIP d248-685, also co-immunoprecipitated with non-muscle myosin IIb (Fig 2). These results confirmed those of our previous study and indicated that the portion of FILIP responsible for its binding to non-muscle myosin IIb is within the 687–960 amino acid region of FILIP [15]. Our results suggest that FILIP-Hsc70 binding is not essential for the binding of FILIP to non-muscle myosin IIb.

### Binding to Hsc70 is important for the effects of FILIP on the subcellular distribution of non-muscle myosin IIb

**Fig 3:**
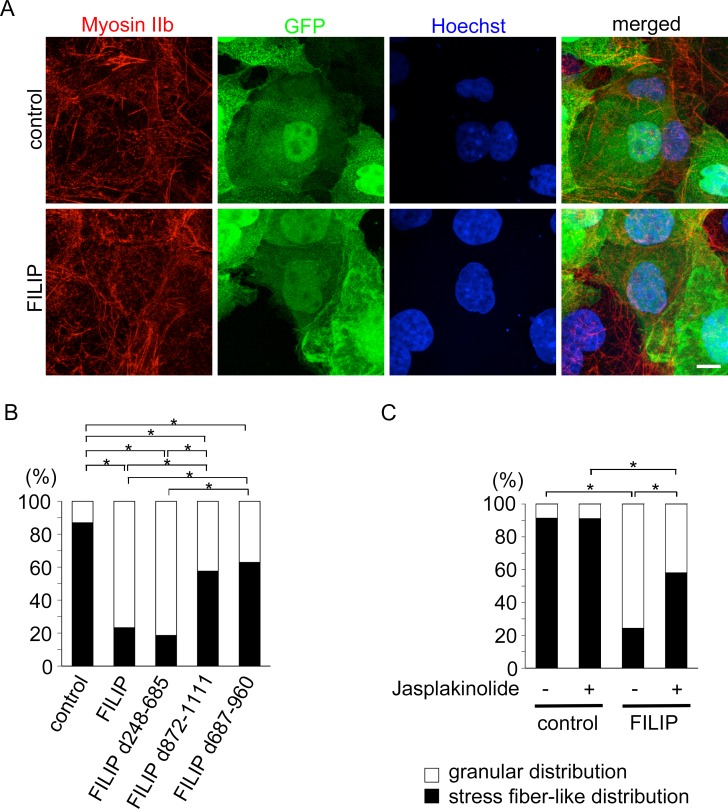
Exogenous expression of FILIP influences the subcellular distribution of non-muscle myosin IIb. (A) Non-muscle myosin IIb was visualized (red) using an anti-NMHCIIb antibody in COS-7 cells. Green, bicistronic GFP expression vector for FILIP; blue, cell nuclei labeled with Hoescht dye. Scale bar = 10 μm. (B) The graph shows the ratio of the cells exhibiting a stress fiber-like distribution of non-muscle myosin IIb. The numbers of cells containing stress fiber-like distributions of non-muscle myosin IIb/total cells were: 287/330 control cells, 74/318 FILIP-expressing cells, 58/312 FILIP d248-685-expressing cells, 186/323 FILIP d872-1111-expressing cells, and 195/310 FILIP d687-960-expressing cells. *p < 0.01 (Fisher’s exact test) (C) The graph shows the effects of jasplakinolide. The numbers of cells containing a stress fiber-like distribution of non-muscle myosin IIb/total cells were: 285/312 control cells treated with vehicle, 286/314 control cells treated with jasplakinolide, 76/313 FILIP-expressing cells treated with vehicle, and 180/310 FILIP-expressing cells treated with jasplakinolide. *p < 0.01 (Fisher’s exact test).

We transfected COS-7 cells with various deletion mutants of FILIP and studied their effects on the subcellular distribution of non-muscle myosin IIb. The effect of the deletion mutant FILIP d687-960, which does not bind to non-muscle myosin IIb, was weaker than that of full-length FILIP (Fig 3A and 3B). In addition, exogenous expression of the deletion mutant FILIP d872-1111, which binds to non-muscle myosin IIb, but not to Hsc70, had a weaker influence than full-length FILIP on the subcellular distribution of non-muscle myosin IIb (Fig 3B). In order to investigate whether the influence of FILIP on the subcellular distribution of non-muscle myosin IIb is dependent on the diminishment of actin fibers, we treated FILIP-expressing cells with jasplakinolide, which is an actin fiber stabilizer. The application of jasplakinolide resulted in the partial rescue of the effect of FILIP on the subcellular distribution of non-muscle myosin IIb (Fig 3C).

### Hsc70 influences the subcellular distribution of non-muscle myosin IIb

**Fig 4:**
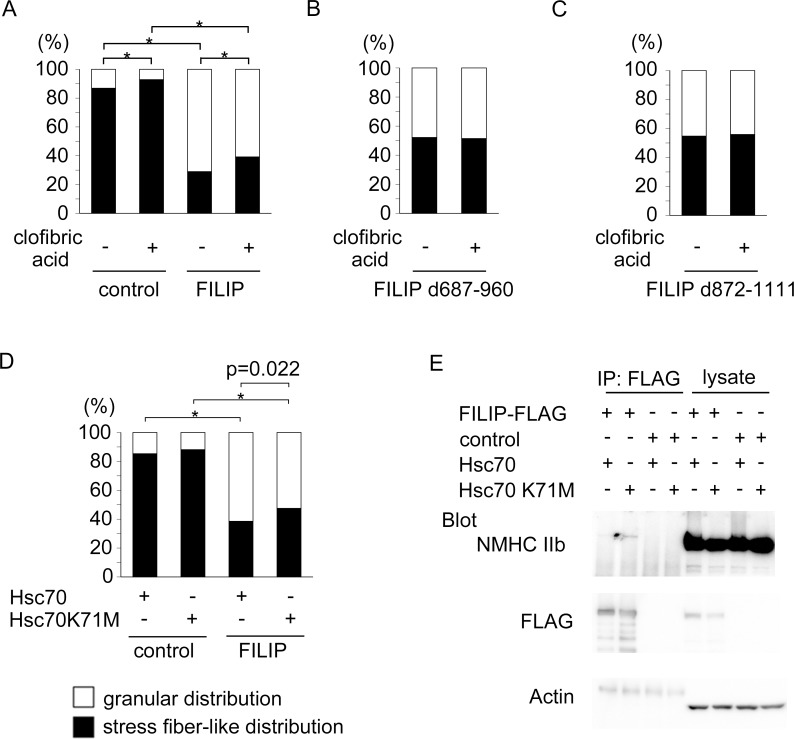
Inhibition of Hsc70 results in the suppression of the effects of FILIP on the subcellular distribution of non-muscle myosin IIb. A-D: The graphs show the ratio of the COS-7 cells exhibiting a stress fiber-like distribution versus a granular distribution of non-muscle myosin IIb (A) after treatment with 2 mM clofibric acid (numbers of cells containing a stress fiber-like distribution/total cells: 543/625 control cells treated with vehicle; 571/615 control cells treated with clofibric acid; 182/628 FILIP-expressing cells treated with vehicle; and 243/620 FILIP-expressing cells treated with clofibric acid. *p < 0.01 (Fisher’s exact test)); (B) under the expression of FILIP d687-960 with or without the application of clofibric acid (numbers of stress fiber-like distributed cells/total cells: 166/318 FILIP d687-960-expressing cells treated with vehicle and 158/307 FILIP d687-960-expressing cells treated with clofibric acid); (C) under the expression of FILIP d872-1111 with or without the application of clofibric acid (numbers of stress fiber-like distributed cells/total cells: 178/325 FILIP d872-1111-expressing cells treated with vehicle and 185/331 FILIP d872-1111-expressing cells treated with clofibric acid); and (D) under the expression of Hsc70K71M and FILIP (numbers of non-muscle myosin IIb stress fiber-like distributed cells 273/320 control/Hsc70-expressing cells; 282/320 control/Hsc70K71M-expressing cells, 131/340 FILIP/Hsc70-expressing cells, and 151/318 FILIP/Hsc70K71M-expressing cells. (E) Mutation of Hsc70 does not influence the binding of FILIP and non-muscle myosin IIb. Immunoprecipitation was performed using an anti-FLAG antibody, and blots were probed with an antibody against non-muscle myosin IIb.

The binding of Hsc70 to its binding partners is controlled by adenosine triphosphate (ATP) or adenosine diphosphate binding at the adenosine triphosphatase (ATPase) domain of Hsc70 [20,21]. In order to investigate whether the ATPase function of Hsc70 is necessary for the alteration of the subcellular distribution of non-muscle myosin IIb, we treated COS-7 cells exogenously expressing FILIP with clofibric acid, which is an inhibitor of the ATPase function of Hsc70 [22–24]. The ratio of the cells that contained a stress fiber-like distribution of non-muscle myosin IIb was increased in the presence of clofibric acid compared to cells exposed to vehicle (Fig 4A). Furthermore, the effects of clofibric acid on non-muscle myosin IIb were not observed in the presence of mutant FILIP unable to bind non-muscle myosin IIb (Fig 4B). These effects were also abolished in cells that expressed a non-Hsc70-binding mutant of FILIP (Fig 4C). We used another inhibitor of Hsc70, VER-155008, which inhibits the function of Hsp70/Hsc70 [25]. Treatment of COS-7 cells expressing exogenous FILIP with VER-155008 had similar effects to treatment with clofibric acid on the subcellular distribution of non-muscle myosin IIb. In addition, the expression of Hsc70 K71M, which is devoid of ATPase activity [26], counteracted the effects of FILIP on the distribution of non-muscle myosin IIb (Fig 4D). In addition, our results suggest that Hsc70 K71M does not inhibit the binding of FILIP to non-muscle myosin IIb (Fig 4E).

### Inhibition of Hsc70 activity in FILIP-expressing neurons results in the inhibition of the elongation of spine length

**Fig 5:**
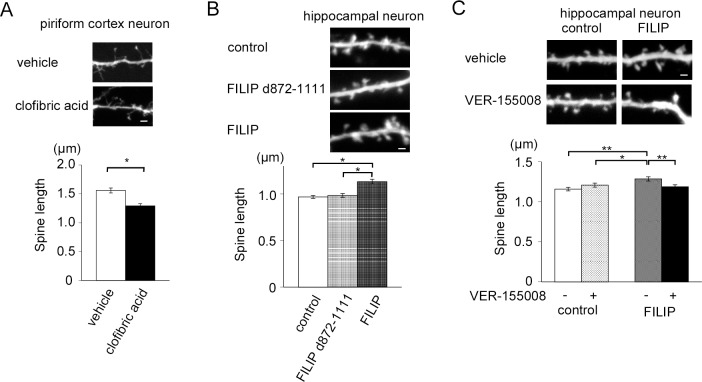
The regulation of spine length by FILIP in primary neurons requires Hsc70. (A) Treatment with clofibric acid resulted in shorter spines in piriform neurons. Scale bar = 2 μm. The graph shows summary data from 554 spines from 6 neurons treated with vehicle and 571 spines from 4 neurons treated with clofibric acid. *p < 0.01 (B) Expression of FILIP d872-1111 in hippocampal neurons did not result in elongated spines. Scale bar = 1 μm. The graph shows summary data from 882 spines from 5 neurons (control), 851 spines from 5 neurons (FILIP d872-1111), and 696 spines from 5 neurons (FILIP). *p < 0.01 (C) Inhibition of the function of Hsc70 reverses the effects of FILIP on spine length. Scale bar = 1 μm. The graph shows summary data from 863 spines from 6 neurons (control/vehicle), 821 spines from 6 neurons (control/VER-155008), 603 spines from 6 neurons (FILIP/vehicle), and 741 spines from 6 neurons (FILIP/VER-155008). **p < 0.01, *p < 0.05.

We previously showed that FILIP controls the morphology of neuronal spines by modulating the activity of non-muscle myosin IIb [15]. Treatment of cultured piriform neurons expressing endogenous FILIP with clofibric acid resulted in shorter spine lengths compared to treatment with vehicle (vehicle, 1.559 ± 0.043 μm, n = 554; clofibric acid, 1.292 ± 0.036 μm, n = 571: Fig 5A). Whereas the exogenous expression of FILIP led to elongation of the spines of hippocampal neurons, as previously reported, the exogenous expression of FILIP d872-1111 did not influence spine length in hippocampal neurons (control, 0.968 ± 0.015 μm, n = 882; FILIP d872-1111, 0.984 ± 0.020 μm, n = 851; FILIP, 1.132 ± 0.026 μm, n = 696: Fig 5B). We investigated whether the influence of the exogenous expression of FILIP on spine length was rescued with VER-155008 treatment. We did not observe an effect of VER-155008 on control neurons that did not express FILIP. However, VER-155008 treatment of FILIP-expressing hippocampal neurons led to significantly shorter spines than vehicle treatment of FILIP-expressing hippocampal neurons. These spines were similar in length to those of control hippocampal neurons (control/vehicle, 1.159 ± 0.022 μm, n = 863; control/VER-155008, 1.209 ± 0.025 μm, n = 821; FILIP/vehicle, 1.287 ± 0.026 μm, n = 603; FILIP/VER-155008, 1.189 ± 0.025 μm, n = 741: Fig 5C). These results suggest that Hsc70 function is important for the elongation of spine length by FILIP.

### FILIP forms dimers in cultured cells

**Fig 6:**
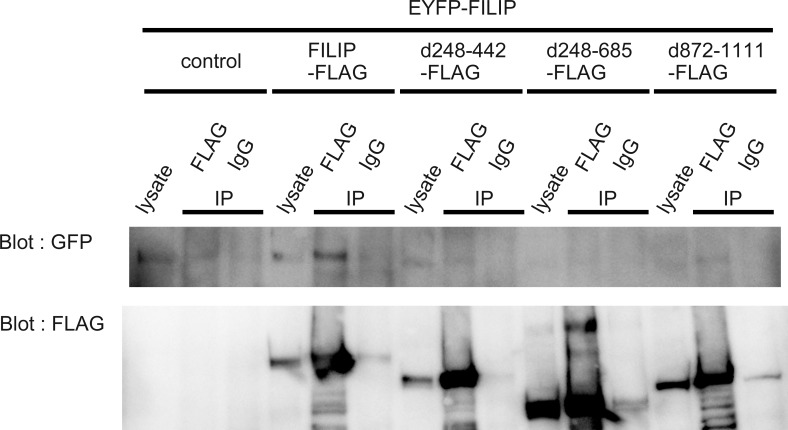
FILIP dimerizes via its SMC_prok_B domain. **FLAG-tagged deletion mutants of FILIP and EYFP-tagged FILIP expression vectors were co-transfected into COS-7 cells.** Immunoprecipitation was performed using an anti-FLAG antibody. FILIP d248-685 did not co-immunoprecipitate with EYFP-tagged FILIP. IgG, normal mouse IgG as a negative control.

A high-molecular weight band was observed on western blots of FLAG-tagged FILIP (FILIP-FLAG)-expressing cells and in immunoprecipitation assays using the anti-FLAG antibody, in addition to a band with the appropriate molecular weight (Fig 2). We hypothesized that FILIP forms homodimers in cultured cells. We co-transfected cells with the FILIP-FLAG expression vector and an EYFP-tagged FILIP (FILIP-EYFP) expression vector. Binding of FILIP-FLAG to FILIP-EYFP was confirmed via immunoprecipitation (Fig 6). We searched for the domain responsible for homodimer formation using several deletion mutants of FILIP. While FILIP d248-442 and FILIP d872-1111 had low affinity for FILIP-EGFP, FILIP d248-685 did not co-immunoprecipitate with FILIP-EYFP (Fig 6). These data indicate that amino acids 248–685 of the FILIP SMC_prok_B domain were responsible for homodimer formation. As the deletion mutant FILIP d248-685 co-immunoprecipitated with non-muscle myosin IIb (Fig 2), dimer formation of FILIP is not crucial for the binding of FILIP to non-muscle myosin IIb.

### The SMC_prok_B domain of FILIP is related to the FILIP-mediated reduction in actin stress fiber content in cultured cells

**Fig 7:**
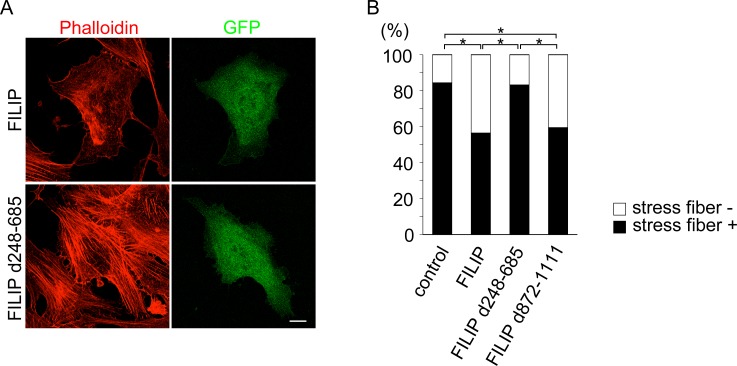
The SMC_prok_B domain of FILIP is responsible for the modification of actin stress fibers. (A) We observed no apparent actin stress fibers in FILIP-expressing NIH3T3 cells. In contrast, actin stress fibers were clearly visible in FILIP d248-685-expressing NIH3T3 cells. Actin fibers were visualized (red) using Alexa 568-conjugated phalloidin. Green, bicistronic GFP expression vector of FILIP. Scale bar = 10 μm. (B) The graph shows the ratio of cells containing actin stress fibers to total cells examined. The ratios were 345/409 for control cells, 233/413 for FILIP-expressing cells, 405/487 for FILIP d248-685-expressing cells, and 208/350 for FILIP d872-1111-expressing cells. *p < 0.01 (Fisher’s exact test).

FILIP expression in cultured cells resulted in a decrease in the proportion of cells containing actin stress fibers (Fig 7A). We wanted to determine the domain of FILIP responsible for actin stress fiber formation in NIH3T3 cells. Whereas the expression of FILIP d872-1111 resulted in a decrease in actin stress fiber formation, the deletion of the SMC_prok_B domain, which is important for FILIP dimer formation, had no apparent effect on actin stress fiber content in these cells (Fig 7A and 7B). Although actin stress fibers consist of actin fibers and actin binding proteins, including non-muscle myosin, our results indicate that the reduction in actin stress fiber formation due to FILIP expression is independent of changes to the subcellular distribution of non-muscle myosin IIb.

## Discussion

We recently reported that FILIP controls the subcellular localization of non-muscle myosin IIb [15]. Here we identify Hsc70 as a binding partner of FILIP and reveal that Hsc70 is involved in the effects of FILIP on non-muscle myosin IIb and the morphology of spines in FILIP-expressing neurons. Hsc70 is a chaperone protein with many cellular functions [27,28]. The expression of Hsc70 is observed in neurons of the central nervous system [29,30], where it has roles in synaptic vesicle recycling and SNARE complex assembly in presynaptic terminals [31–33]. Hsc70 is also present in postsynaptic sites [29]. Our results suggest a new function for Hsc70 at postsynaptic sites and indicate that Hsc70 is important for postsynaptic neurotransmission. Recently, Hsc70 has been shown to be involved in protein misfolding diseases, such as Alzheimer’s disease, and Parkinson’s disease. In fact, Hsc70 is considered to be a potential therapeutic target in these diseases [34–37]. Treatment of a mouse model of tauopathy with an inhibitor of Hsc70 results in the reduction of aberrant tau levels the rescue of synaptic plasticity deficits [37]. It is possible that the rescue of synaptic plasticity deficits in this tauopathy model following the administration of the Hsc70 inhibitor involves the mechanism described in this study.

**Fig 8:**
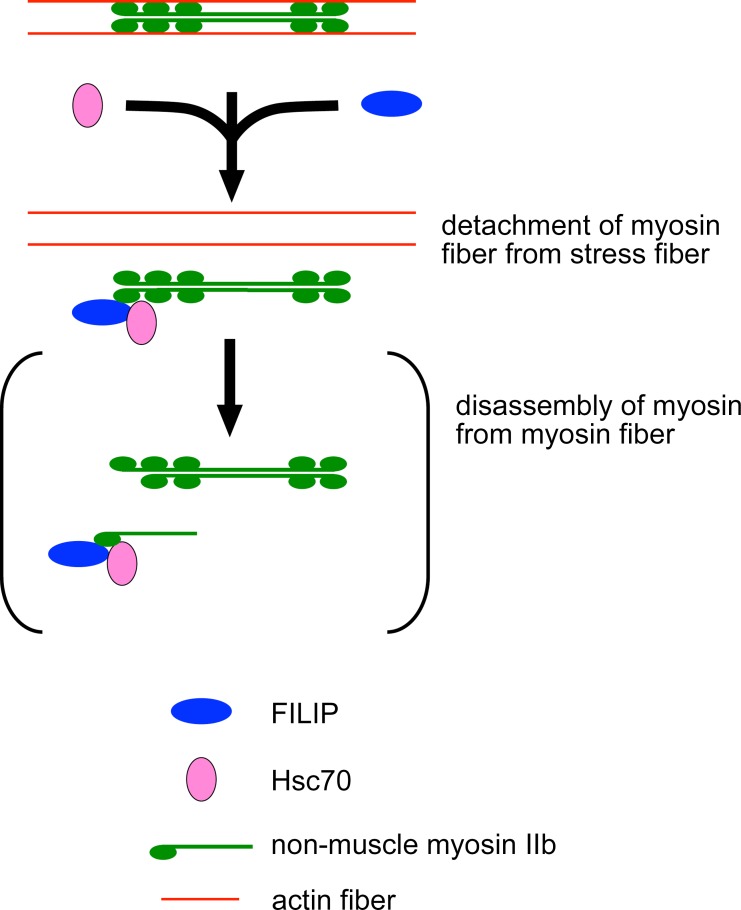
Schematic illustration of the effects of FILIP and Hsc70 on non-muscle myosin IIb.

Interactions of Hsc70 with substrate proteins are necessary for its function [27,28], which is regulated by ATP hydrolysis [28]. Here we show that the ATPase function of Hsc70 is involved in the dissociation of non-muscle myosin IIb from actin stress fibers in mammalian cells. This suggests that the formation of a protein complex of Hsc70, FILIP, and non-muscle myosin IIb promotes the dissociation of non-muscle myosin IIb from actin fibers. As one of functions of Hsc70 is to help disrupt multiprotein complexes [28], we postulate that Hsc70 helps with the disassembly of non-muscle myosin IIb from myosin fibers (Fig 8).

Inhibition of non-muscle myosin II function leads to a decrease in stress fiber formation [38,39]. However, the current data on the effects of FILIP on the distributions of non-muscle myosin IIb and actin stress fibers suggest that the detachment of non-muscle myosin IIb from stress fibers in the presence of FILIP does not directly lead to a reduction in the levels of thick actin fibers. As COS-7 cells contain both non-muscle myosin IIb and IIc [40], there is a possibility that non-muscle myosin IIc compensates for the function of non-muscle myosin IIb in the presence of FILIP. Although we could not exclude this possibility, our data indicate that levels of thick actin fibers do not decrease at a rate proportional to the dissociation of the non-muscle myosin IIb from the actin fibers. An indirect effect of FILIP on the decrease in the levels of actin stress fibers is also supported by our data on the effects of FILIP d248-685 on the subcellular distributions of non-muscle myosin IIb and actin fibers. Nevertheless, as jasplakinolide is an actin fiber stabilizer [41], our data on the application of jasplakinolide indicate FILIP directly affects non-muscle myosin IIb. Our results suggest that FILIP has two effects on the actomyosin complex: the dissociation of myosin IIb from actin stress fibers, and the attenuation of actin fiber formation.

In conclusion, we have identified the chaperone protein Hsc70 as a new binding partner of FILIP and demonstrate that the interaction between Hsc70 and FILIP is essential for FILIP-induced changes in non-muscle myosin IIb and the morphology of spines in FILIP-expressing neurons. FILIP appears to have complex effects on non-muscle myosin IIb, including direct effects via an interaction with Hsc70 and indirect effects via its impact on actin stress fibers.

## Supporting information

S1 Fig Schematic drawing of full-length and deletion mutants of FILIP. Black box: SMC_prok_B domain. Gray box: Herpes_BLLF1 domain.
(TIF)

S2 Fig Hsc70 is a candidate binding protein for FILIP. COS-7 cells were transfected with FLAG tagged FILIP or the control vector. Immunoprecipitation was performed using an anti-Hsc70 antibody. IgG, normal rat IgG used as a negative control.
(TIF)
