# Supplementary figures and images for: Subcellular distribution of non-muscle myosin IIb is controlled by FILIP through Hsc70
Source: PLoS One. 2017 Feb 24;12(2):e0172257. doi: 10.1371/journal.pone.0172257 (PMC5325215; doi:10.1371/journal.pone.0172257)

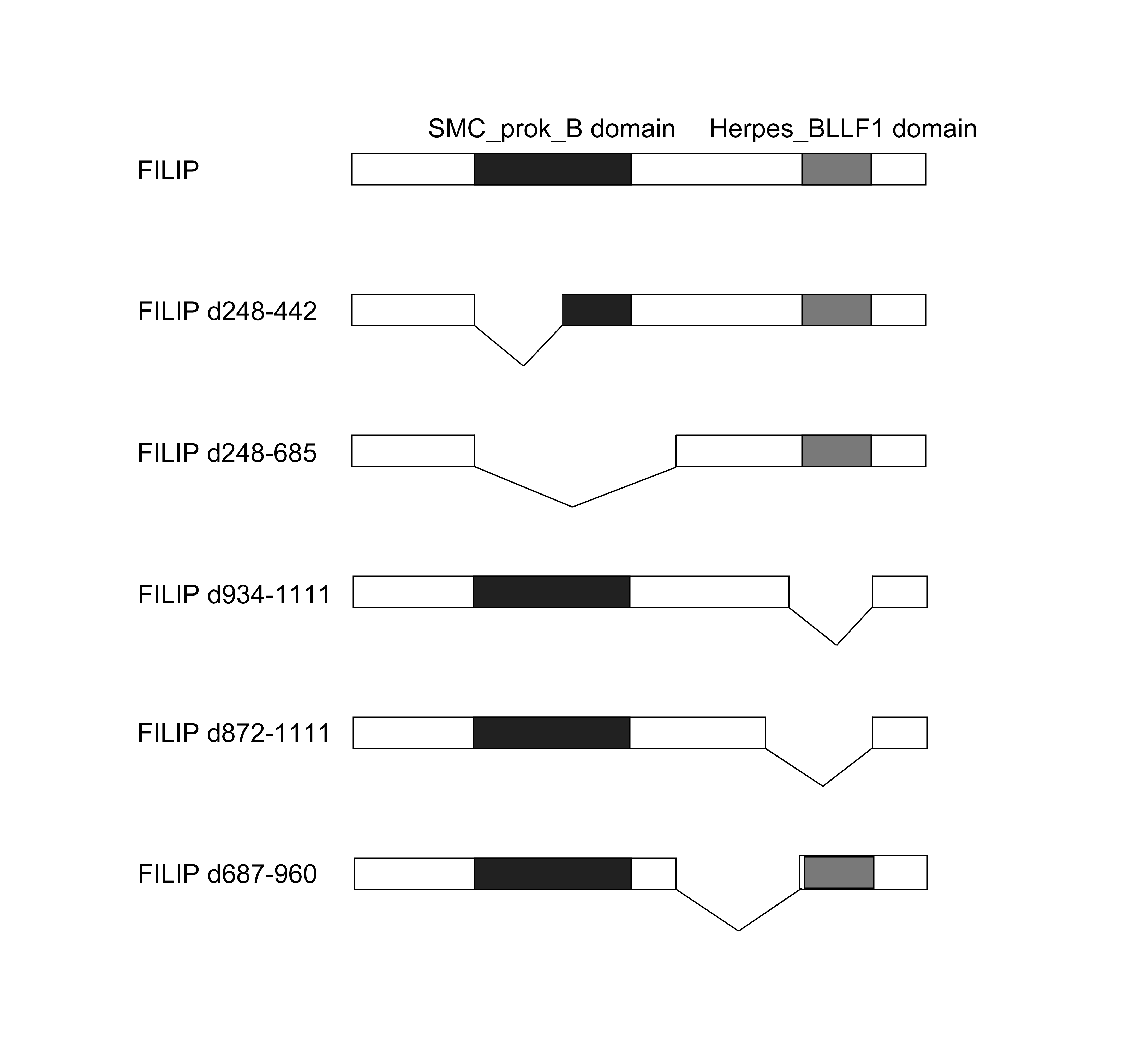

Supplement: S1 Fig — Black box: SMC_prok_B domain. Gray box: Herpes_BLLF1 domain. (TIF) [file pone.0172257.s001.tif]

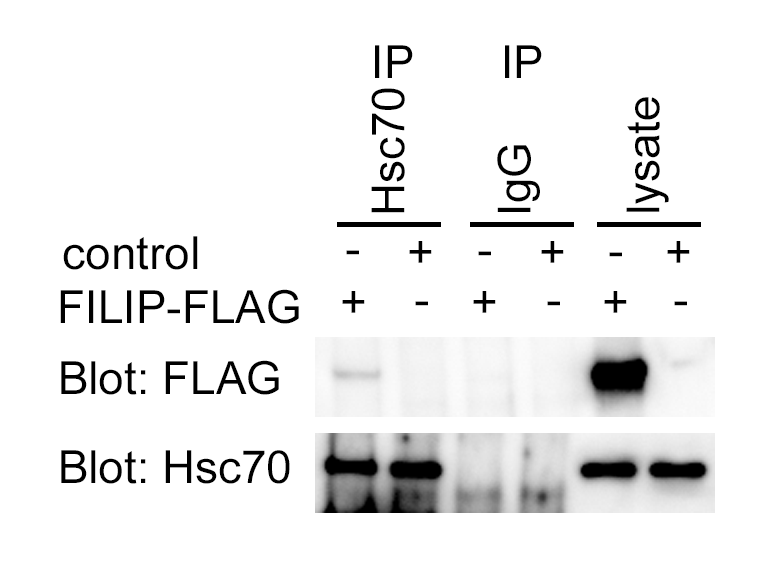

Supplement: S2 Fig — COS-7 cells were transfected with FLAG tagged FILIP or the control vector. Immunoprecipitation was performed using an anti-Hsc70 antibody. IgG, normal rat IgG used as a negative control. (TIF) [file pone.0172257.s002.tif]
